# Sexual dimorphism in the horn size of a pair-forming coral reef butterflyfish

**DOI:** 10.1371/journal.pone.0240294

**Published:** 2020-10-08

**Authors:** Satoshi Shiratsuchi, Chancey MacDonald, Maya Srinivasan, Geoffrey P. Jones

**Affiliations:** 1 ARC Centre of Excellence for Coral Reef Studies, College of Science and Engineering, James Cook University, Townsville, QLD, Australia; 2 California Academy of Sciences, San Francisco, CA, United States of America; Institut de recherche pour le developpement, FRANCE

## Abstract

Sexual dimorphism is a common in the animal kingdom and is often linked to mate choice or competition for mates in polygynous mating systems. However, sexual dimorphism is less common in species that form heterosexual pairs and has not been recorded in pair-forming coral-reef fish. Here we demonstrate a pronounced morphological difference between males and females in the humphead bannerfish (*Heniochus varius*)—a pair-forming coral reef butterflyfish. Males of paired individuals collected in Kimbe Bay, Papua New Guinea had substantially larger hump and horn protrusions on their heads than females. Fish were also sexed, sized and aged to determine the reproductive and demographic basis of the pairing behaviour. *H*. *varius* pairs were exclusively heterosexual and were assorted strongly by total length and slightly less so by age. Females in pairs were generally the same size as male partners, but were frequently older by a year and sometimes more. Hump and horn lengths increased proportionally to body-size in both sexes, with horns growing at a greater rate among males. These findings suggest that *H*. *varius* form pairs primarily for reproductive purposes, with selection via a size-assortative process that likely also extends to selection for larger hump and horn protrusions among males. The larger humps and horns in males appear to be the first recorded example of a secondary sexual morphological characteristic in a pair-forming coral reef fish species.

## Introduction

In sexually reproducing animals, fitness is contingent upon finding mates, successful reproduction and the contribution of offspring to the next generation [1–3]. Animal mating systems may involve reproduction via one or several sexual partners and the system adopted is often related to levels of parental care and other traits such as body size and the level of physical differentiation between sexes [4]. For example, monogamous mating systems, the prolonged and exclusive mating association between a male and a female pair, are commonly associated with sexual monomorphism or higher levels of biparental care [4,5]. In contrast, sexual dimorphism is more common in species that invest little in parental care and engage in polygamous mating systems [6–9]. Some species present a challenge to this rule by appearing to have a monogamous mating system, despite having no biparental care and in some cases exhibiting sexual dimorphism in colour, e.g. [10–12]. Studies of these exceptions can further our understanding of natural and sexual selection in the evolution of mating systems.

Pair formation and presumed monogamy appear to be unusually prevalent in coral reef fish [13,14]. Almost 350 pair-forming species are documented from 29 coral reef fish families (~17% of reviewed species) [13]. As most coral reef fish exhibit little or no parental care of offspring, the underlying reasons for prolonged pair formation are uncertain. Proposed drivers include increased reproductive efficiency [14–17], foraging cooperation [13,18–24], and/or mutualistic resource/territory defense [13,14,18,24–27]. Whilst some of these benefits may be achieved via either heterosexual or homosexual pairs, pair formation that is directly related to reproductive efficiency should result in higher rates of pairing among adult fish than juveniles and higher rates of heterosexual than homosexual pairing. Though pair bonding is not always a reliable predictor of reproductive monogamy (e.g. [28]), identifying the potential drivers of pair formation within a species first requires determination of the sex and maturation status of individuals in pairs.

Size assortative pairing (pairs of similar sized individuals) is also common among pair forming coral reef fish, including those exhibiting both homosexual and heterosexual pairs [1,25,29–35]. Size-assortative heterosexual pair formation for reproductive purposes, or ‘size assortative mating’, often results from both sexes pursuing larger mates in order to maximize reproductive output [1]. Males are likely to prefer larger females because they have higher fecundity and females may prefer larger males because they are likely to contribute more to joint defense of critical resources or are better able to ward off predators [1,5,12,36,37]. Size-assorted homosexual pairs may arise if same-sex individuals reform pairs in the process of competing for larger partners. However, strong relationships between both the sizes and ages of individuals in any pair may indicate the long-term maintenance of pairs formed at early life stages, rather than size assortative mating *per se* [12]. Understanding the presence and prevalence of size-assortative mating and the sexual basis of pairing is essential in understanding the purpose of pairing behaviours in coral reef fish.

Pair formation is generally associated with sexual monomorphism and weaker sexual selection across the animal kingdom, including among reef fish [10,38,39]. In contrast, sexual dimorphism, and in particular, morphological characteristics such as horns and antlers are more prevalent in males of species with polygamous mating systems, where males fight to compete for multiple females [40–44]. In mammals, horns are often absent in females, but when they do occur, they are often smaller in females than in males [43]. Larger horn size in males may result from sexual selection [45]. For example, in North American elk and European red deer, larger antlers indicate greater strength, power and overall higher fitness, and therefore females prefer to mate with males with larger antlers [46]. The occurrence of sexual dimorphism in pair-forming species is relatively rare and is undocumented among reef fish but may provide clues as to why species form pairs.

Butterflyfishes (family Chaetodontidae) are a useful group to examine the underlying basis of pairing behaviour. This family is highly conspicuous on coral reefs and over 60% of the 93 current species form pairs, with both heterosexual and homosexual pairing documented [12,13,33,47–51]. Furthermore, pair bonds in other species of butterflyfish have been recorded to last up to 10 years [51,52]. While the mating system is assumed to be monogamous [33,53–56], this has rarely been established (but see, [57]). Like most other pair-forming reef fish species, butterflyfish lack biparental care [10–12] and to date, no sexual dimorphism has been documented [10]. However, our preliminary observations of the humphead bannerfish (*Heniochus varius*) suggested a predominant group size of two individuals and morphometric differences between paired fish in the shape of unique head ornamentation that may represent the first example of sexual dimorphism in a seemingly socially monogamous pair-forming coral reef fish.

The aims of this study were to investigate the ratio of heterosexual pairing *H*. *varius*, to evaluate the evidence for size and age assortative pairing, and to determine whether the size of hump and horn protrusions represent secondary sexual characteristics or sexual dimorphism in the species. Firstly, we characterize the general age and size structure of the sample population. We then hypothesize that pairing is related to mating by testing the predictions that: (1) all or most pairs should be heterosexual, and that (2) pairing is size and age assortative, with correlations between females and their male partners. Finally, we test whether (3) humps and horn protrusions on the head of *H*. *varius* are secondary sexual characteristics—being larger in males than their female partners.

## Materials and methods

This study was conducted on nine inshore reefs in Kimbe Bay, West New Britain, Papua New Guinea (5^o^S, 151^o^E), between March and May of 2015 (S1 Fig). These platform reefs varied in steepness from shallow-water reef slopes to steep-walled reefs.

### Sample collection

A total of 76 individual *H*. *varius* were collected using low-powered spear guns, between 1 m and 18 m depths. The sample included both individuals in 25 known pairs, and 26 individuals from known pairs where only one member was successfully collected (total n = 39 females and 37 males). Fish were observed for several minutes prior to collection to determine that they were paired. Paired fish move together in close association (usually <1m apart) and do not interact aggressively toward one another. All adult fish observed in the field were paired.

### Ethics statement

Fish handling and sampling was conducted in accordance with the Australian Code for the Care and Use of Animals for Scientific Purposes, 8th Edition, 2013, Queensland Animal Care and Protection Act, 2001, and the Australian Guidelines for the Humane Killing of Animals used for Scientific Purposes. Sampling methods were approved by the James Cook University Animal Ethics Committee (Ethics Approval A2165), under the James Cook University Animal Ethics Committee Terms of Reference in relation to the above national and state Codes and Acts. All sampled fish were euthanized with a 1:4 clove oil/ethanol solution as soon as they were caught and immediately placed in an ice bath after collection. All efforts were made to streamline the speed of the process, to minimize animal suffering. This research was approved under a special exemption researcher/academic permit (10350006662), issued by the government of Papua New Guinea. Verbal permissions were granted by Mrs. Cecilie Benjamin (Chair of the Board, Mahonia Na Dari Research and Conservation Centre, Kilu) and Mr. Thomas Koi, (Village Elder and representative of the Local Marine Management Committee, Kilu).

### Sample measurements

**Fig 1:**
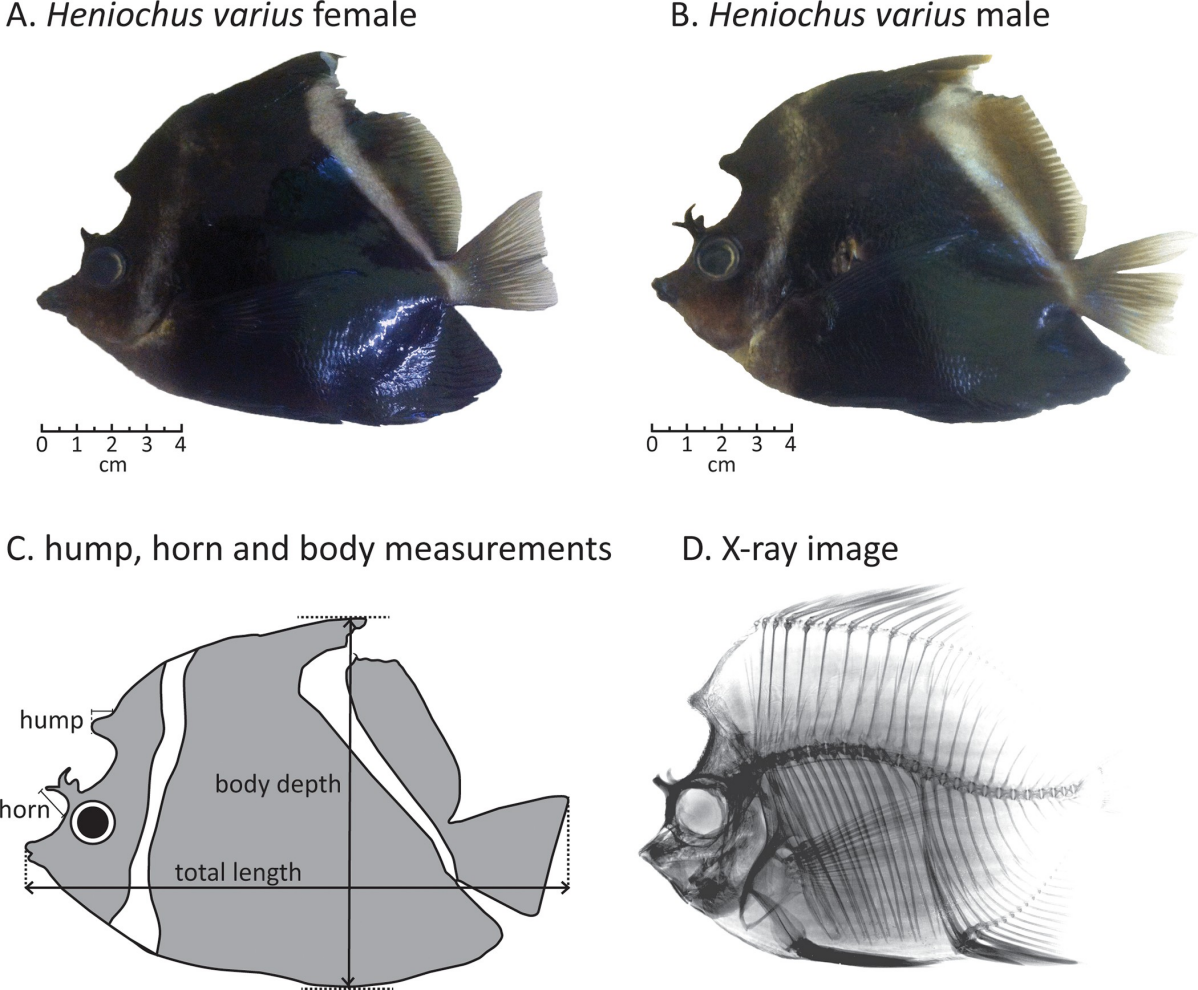
Diagrams of *Heniochus varius* illustrating their cranial hump and horn structures. The first two images show sexual dimorphism between the female (A) and male (B) within a size assorted breeding pair. (C) Shows the size measurements taken. (D) Shows an X-ray image illustrating the internal structures of the hump and horn features.

Following collection, individuals were measured, weighed and dissected. Total length (TL), body depth, hump length and horn length (Fig 1) were measured to the nearest millimeter using calipers. Wet weight was measured to the nearest gram and the gonads and sagittal otolith were extracted from each individual post weighing. The gonads were fixed in formaldehyde (4%)–acetic acid-calcium chloride (FACC) and the otoliths were cleaned after dissection and stored dry. To confirm the sex, either the whole gonad (if < 5 mm in length) or a transverse section of the gonad (if > 5 mm) were used. Determining the sex of the sample followed the same procedure as the study conducted on *C*. *multicinctus* in Tricas & Hiramoto [58]. Gonads were dehydrated in ethanol and equilibrated in xylene, embedded in paraffin wax and sectioned to 5μm using a microtome. The samples were mounted onto a glass slide and then stained using Mayer’s Haemotoxylin and alcoholic Eosin. Oocytes and sperm tissues were observed under the microscope to determine the sex of the individual (S2 Fig). The age of each fish was estimated by counting sagittal otolith growth rings, following the assumption that these represent annuli as in other Chaetodontidae species [59] and other reef fish species at the same location (Srinivasan *unpublished data*). The otoliths were sectioned and mounted onto glass slides to determine the age of individual fish following Choat & Axe [60] and Hart & Russ [61]. The sectioned otoliths were read using a dissecting microscope at 30~40X magnification with transmitted light. Annuli were counted from the nucleus to the proximal surface of the sagitta, along the ventral margin of the sulcus acousticus (S3 Fig), as previously used for *Chaetodon larvatus* [59] and *Pomacentrus moluccensis* [62].

### Data analysis

All analyses were conducted in R version 3.5.2 [63]. Linear models were assessed in the package ‘lme4’ [64], model fits were assessed for gross departures from normality and homoscedasity of residuals, using model assessment plots in the package ‘performance’ [65]. The same package was used to calculate R^2^ values. We used model component effect estimates and their 95% confidence intervals to assess and report statistically significant factors in linear models; i.e. where the interval between the lower confidence limit (LCL) and the upper confidence limit (UCL) did not cross zero. Where log-scaled predictors were used, model fits were back transformed (exp) in order to present plots on natural scales. Correlations were assessed using ‘cor.test’ in the R base package ‘stats’ [63].

To examine the general size and age structure of the sample population we analyzed two linear models with interaction terms. The first model assessed variation in total length as a product of increasing age. The second model assessed the relationship between wet weight and total length. Both models allowed for different relationships among males and females. Age was log transformed in order to meet the assumptions of normality in model residuals.

To assess size and age assortation in pairing, total length, body depth, wet weight, and age were correlated between males and females, among paired members, using Pearson’s product moment correlation. Because body depth and mass can be allometric with total length, size corrected body depth and wet weight measures were created by regressing each metric against log (TL) and retaining the residuals. These residuals were then also tested for correlations between the sexes of paired individuals.

The assessment of sexual dimorphism and sexual differences in allometric growth of hump and horn protrusions used a subset of total sampled individuals, as fish ≤ 81 cm had not yet developed measurable head protrusions. For within-pair assessments, we investigated protrusion length differences within 24 pairs and for the wider population we assessed relationships among and between 34 males and 37 females.

For within-pair assessments of sexual dimorphism, the length of protrusions was first standardized for individual body length by dividing protrusion length by total length. Linear mixed-effect models were used to compare standardized protrusion length between sexes, with pair identity utilized as a random grouping factor. The relative length of each protrusion feature was also compared between male and female pair members using correlations of residual protrusion lengths. Residual protrusion lengths were obtained by first modelling protrusion lengths against total lengths, using the same linear model approach as for body depth and wet weight (above) and extracting the model residuals.

We investigated sex related variation in the allometric growth of head protrusions by modelling the length of protrusions for each individual in the usable sample population against total length, with sex as a multiplicative covariate. Relationships between the lengths of humps and horns within individuals were also assessed, using a Pearson’s product-moment correlation of the overall sample population, for each sex.

## Results

### General size and age structure

**Fig 2:**
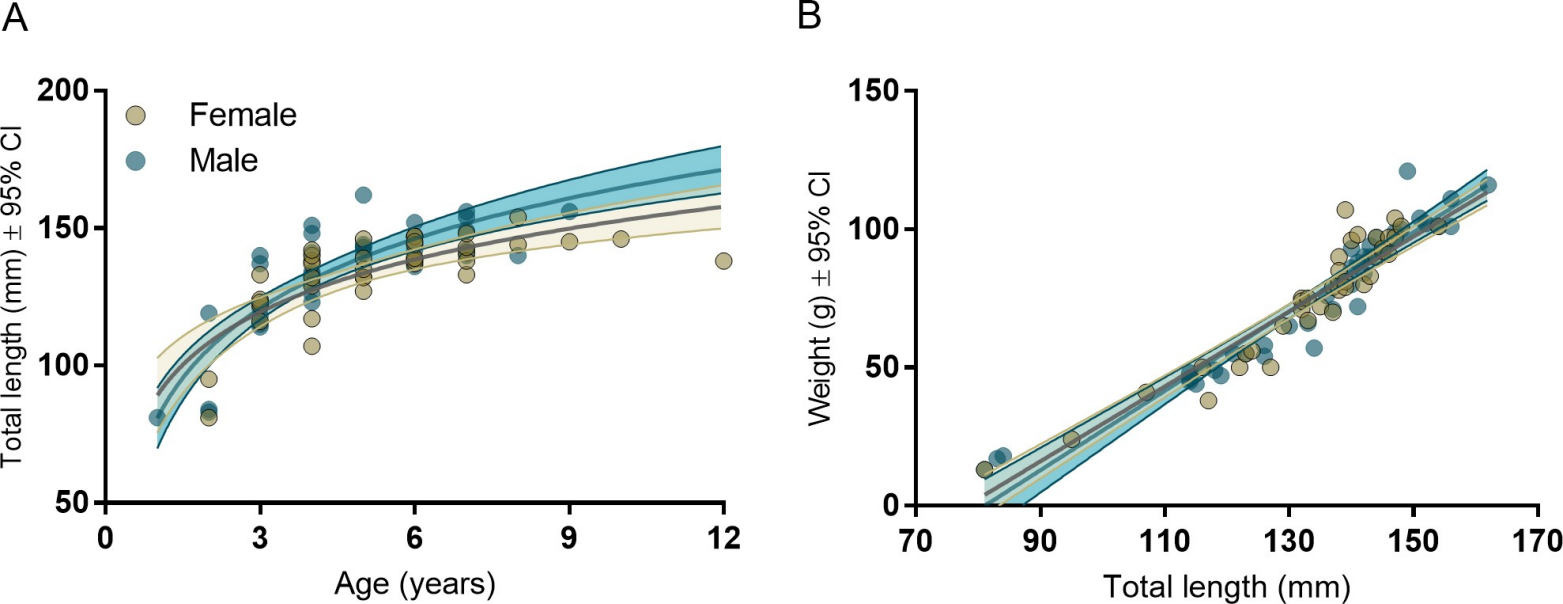
(A) Length vs Age and (B) Weigth vs Length relationships for 76 individual *Heniochus varius* (25 pairs as well as 26 single individuals where the other member of the pair was not caught). Males are dark blue and females are gold.

The total length of *H*. *varius* increased with (log) age (Adjusted R^2^ = 0.64, TL estimate = 0.23, LCL = 0.016, UCL = 0.30) and weight increased with total length (Adjusted R^2^ = 0.90, TL estimate = 1.44, LCL = 1.26, UCL = 1.61), in the total population sample. However, there were no sex related differences in these relationships (Fig 2) (length-age: sex; LCL = -5.86, UCL = 6.57; interaction term; LCL = -1.38, UCL = 1.12; weight-length: sex: LCL = -19.46, UCL = 39.87; interaction term; LCL = -0.30, UCL = 0.14).

### Size and age assortative heterosexual pairing

**Fig 3:**
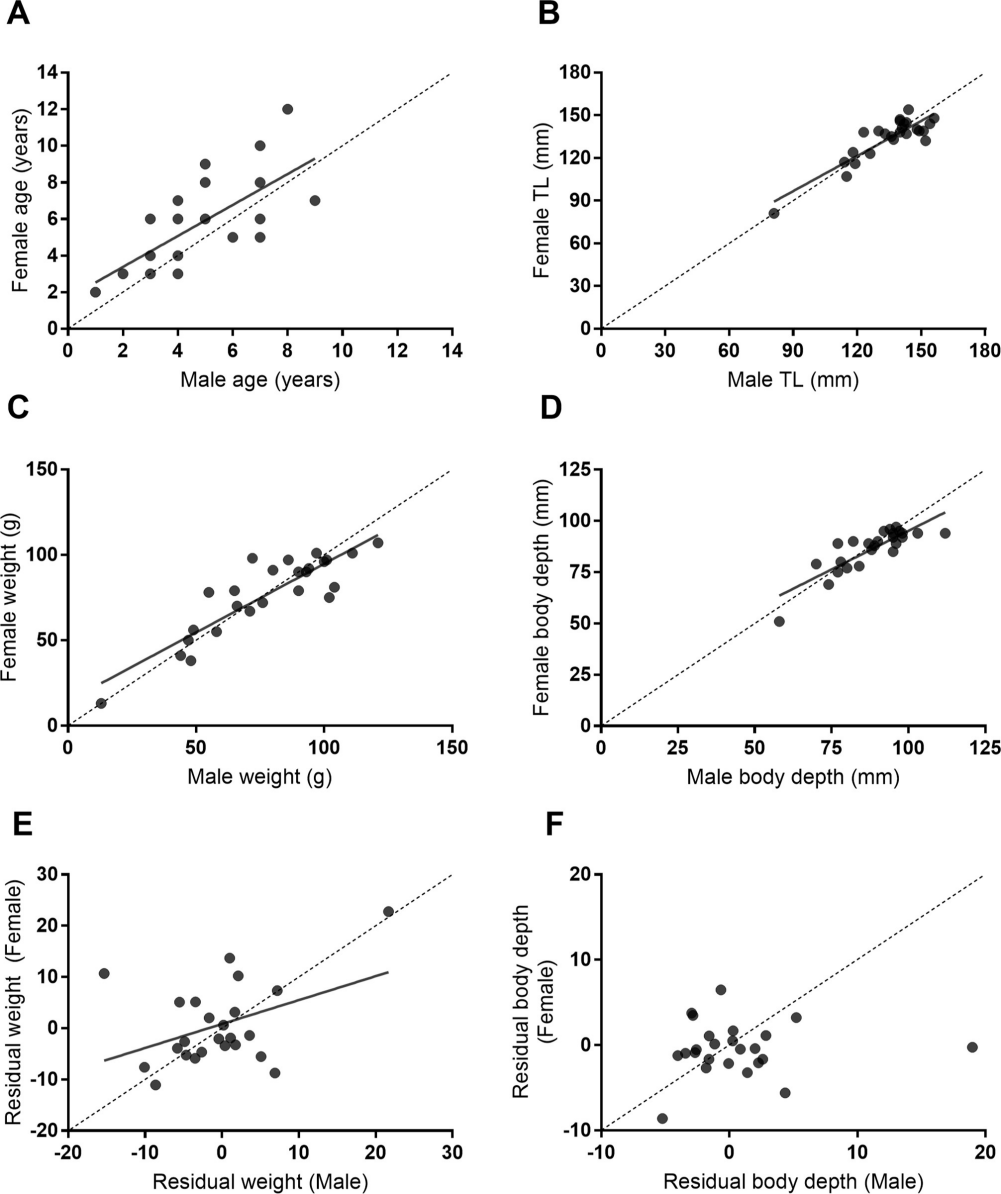
Correlations between males and females for: (A) age, (B) total length, (C) raw mass, and (D) raw body depth, in 25 pairs of *Heniochus varius*. Residual mass (E) and body depth (F) relationships are also shown, after accounting for relationships in the raw metrics to total length. Each point represents a pair, dotted lines indicates line of equality or the expected point if the male and female had an identical metrics, and solid lines represent linear estimates for statistically significant correlations (alpha = 0.05).

Visual and histological examination of gonads confirmed all 24 pairs were heterosexual. Females were characterized by early stage oocytes and males by primary sperm tissue (S2 Fig). All three size variables were strongly positively correlated between partners (Fig 3), i.e. total length (r = 0.88, *t* = 8.84, *p* < 0.0001; Fig 3A), body depth (r = 0.84, *t* = 7.47, p < 0.0001; Fig 3B) and wet weight (r = 0.88, *t* = 8.88, *p <* 0.0001; Fig 3C). After controlling for variation in total length, wet weight remained moderately correlated between the pairs (r = 0.42, t = 2.15, p = 0.042), but body depth did not (r = 0.06, t = 0.258, p = 0.799). This suggests that pairing is size assortative in relation to total length and mass. The age of partners was also positively correlated among the pairs, although more weakly than for total length (r = 0.69, *t* = 4.59, *p* < 0.001; Fig 3D). Females were older than males in 14 pairs (58%), whereas males were older in only 6 pairs (24%), with females being on average 0.96 years older than their male partner. Individuals in the remaining pairs were of the same age.

### Sexual dimorphism and allometric growth

**Fig 4:**
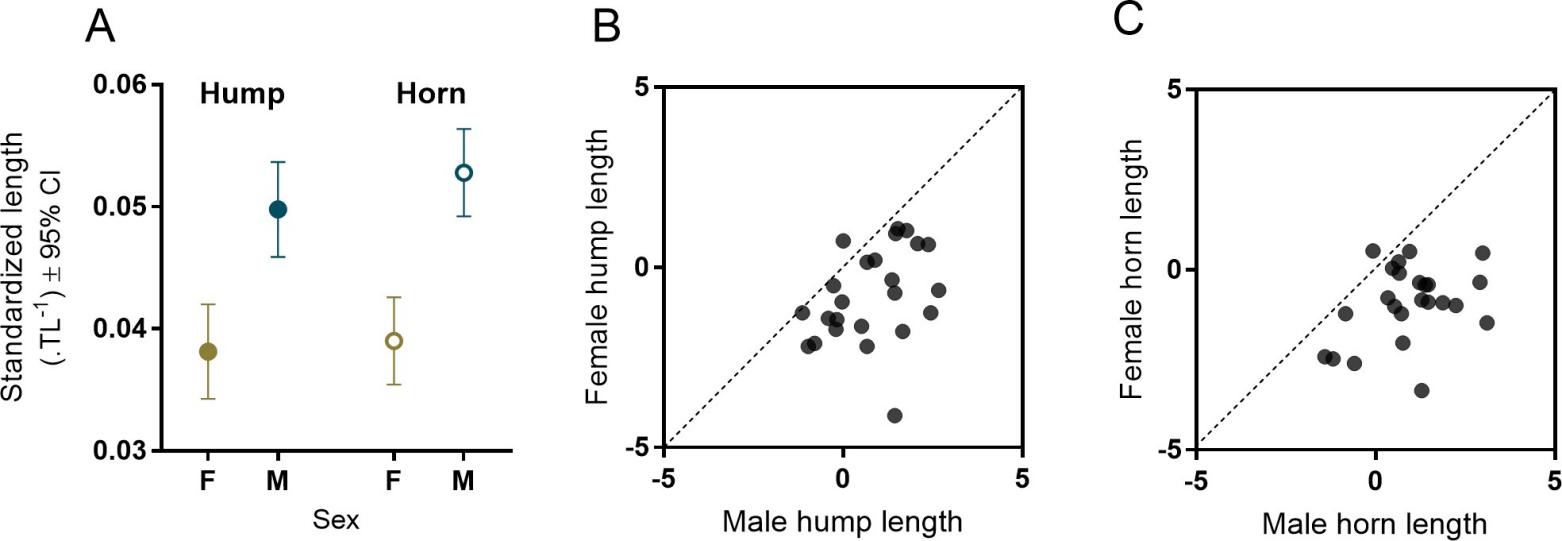
(A) Differences in hump and horn lengths standardized by total body length, for males and females in 25 pairs of *Heniochus varius*. Within-pair intersex comparisons of residual (B) hump and (C) horn length, after accounting for relationships to total length. Dotted lines are lines of equidistance.

**Fig 5:**
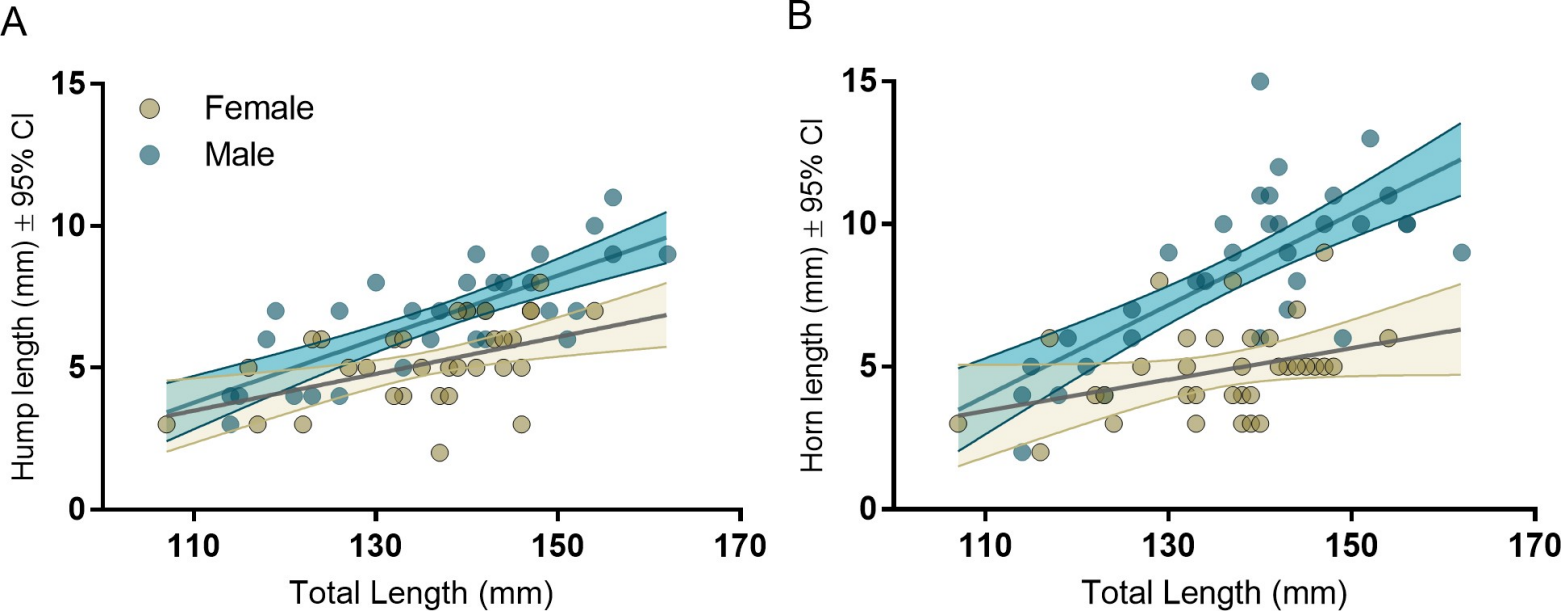
Sex differences in the relationships between total length and (A) hump length, and (B) horn length of 76 *Heniochus varius* individuals sampled in Kimbe Bay, Papua New Guinea. Females are gold and males are dark blue.

Sexual dimorphism in the length of horn protrusions was evident both across the sample population and within pairs. However, while hump length was also dimorphic among paired individuals, there was greater variation in horn length differences between males and females of a given body length. Within partners, mean protrusion length, relative to body size (TL), was significantly longer on males than on their female partners (hump: estimate = 0.012, LCL = 0.007, UCL = 0.016; horn: estimate = 0.014, LCL = 0.010, UCL = 0.018; Fig 4A). In real terms, the average difference was 1.6 mm (24%) longer for hump length and 3.4 mm (42%) longer for horn length. Horns were longer on males in all but one pair (96%) and male’s humps were longer in all but three pairs (88%) (Fig 4B and 4C). Both hump length and horn length increased with body length in both sexes of the overall sample population, the rates of increase were higher among males than females, in both cases (Fig 5) (Hump: Adjusted R^2^ = 0.55, p < 0.001; sex; LCL = -11.96, UCL = 2.13; TL, LCL = 0.02, UCL = 0.11; Interaction term; estimate = 0.11, LCL = 0.03, UCL = 0.18; Horn: Adjusted R^2^ = 0.64, p < 0.001; sex, Interaction term; estimate = 0.11, LCL = 0.03, UCL = 0.18). Further, after controlling for body length, neither hump length nor horn length were correlated between sexes, across the entire sample population (Horn: r = 0.35, p = 0.096; Hump: r = 0.36, *p* < 0.088). Lastly, hump length and horn length were moderately correlated to each other within individual males (r = 0.63, t = 4.58, p < 0.001) but not within females (r = 0.01, t = 0.06, p < 0.954).

## Discussion

The larger hump and horn sizes in male *H*. *varius* represent the first example of external sexual dimorphism in the family Chaetodontidae, and in fact, for any pair-forming coral reef fish species. Measurements of head protrusions revealed a distinct sexual dimorphism within pairs, with 96% of males having longer horns and 88% having longer head humps than their female partner. Head protrusions first appeared at ~90 mm and grew allometrically among both sexes, but at a greater rate among males. The results also show the bannerfish *H*. *varius* pairs are exclusively heterosexual and are likely based on size-assortative mating strategies. This is the first recorded example of a secondary sexual morphological characteristic in a paired (and potentially monogamous) coral reef fish species. Therefore, *H*. *varius* likely represents a deviation to the perception that species forming heterosexual pairs should be sexually monomorphic [4], as particularly evidenced among reef fishes. The functions of the hump and horn remain unknown but based on these results, we propose that within-pair sexual dimorphism of these unique morphological features is likely related to selection for larger head protrusions on males.

The finding of sexual dimorphism in a pairing species is rare in fishes, as well as in broader animal groups [10,38,39], and provokes questions about its evolutionary drivers. The exclusive heterosexual pairing and strong size assortment of males and females support the hypothesis that there are reproductive benefits for pair formation in *H*. *varius* [1]. Traditional interpretations would suggest the strongly heterosexual social parings are also directly related to reproductive monogamy [4,5]. However, this hypothesis is not always supported by molecular evidence, which shows extra-pair fertilizations can be common in a seemingly monogamous species (e.g. [28]). Therefore, sexual dimorphism here may also alternatively suggest that *H*. *varius* is subject to sexual selection—potentially supporting polygamy or partner sorting—despite exhibiting a predominantly pairing social system.

The similar age differences in many pairs potentially suggests a level of longevity in partnerships. For example, in the Greater flamingo, age-assortative pairing occurs when pairs form at a young age, there is a high survival rate, so pairs stay together throughout their life [66]. However, despite being closely size matched, female *H*. *varius* were often at least one year older than their male partner and the largest age difference between partners was four years. Therefore, later pair sorting may also occur, and our results suggest that size-assortative selection is a stronger characteristic of pair formation than age in this species.

Despite the close size matching in total body length, males consistently had larger head protrusions than their female partners. The magnitude of difference in hump and horn length between males and females also increased with body size. Interestingly, while hump length appeared to increase proportionally to body size in both sexes, horn length increased at a greater rate among males. Additionally, lengths of the two head protrusions were moderately correlated among males, but not females. Therefore, we propose that greater horn length may signal greater sexual fitness among males–as is the case among a diverse range of organisms, from Dung beetles to Roe deer, e.g. [42,45]. If females select size matched, but often younger, males in size-assortative mating, we propose this may also extend to selecting males with larger horns as secondary sexual characteristics [46]. Likewise, if males are competing for larger females, these secondary sexual characteristics may enhance the effectiveness of dominance displays, e.g. [39,42,45].

Secondary sexual morphological characteristics such as horns and antlers are generally considered to play a role in dominance displays and/or physical aggression related to the defense of partners, territories and resources [41,42,44]. While our study provides evidence of within pair sexual dimorphism in head-protrusion size in *H*. *varius*, it remains unknown whether larger male head protrusions influences dominance structures directly or if humps and horns are utilized in occasional aggressive interactions. Territorial behaviours are reasonably common among pair-forming butterflyfish species, e.g. [24–27,33,51,53,67]. While much less common, examples of prolonged aggressive interactions, including mouth wrestling, have also been observed among multiple butterflyfish species, which occasionally result in injury bad enough to cause likely death [68–70]. While no direct contact has been observed in the aggressive displays of *H*. *varius*, anecdotal evidence from this study suggests antagonistic interactions (including prolonged chases) are relatively common.

The larger horns in male members of heterosexual pairs may of course have other secondary benefits that are not directly related to antagonistic behaviours. Among ungulates, female horns are often smaller and can be shaped differently to males, which has led to the consideration of different functions of horns in males and females [43]. For *H*. *varius*, larger horns may substitute daily energy expenditure on territorial defense, if larger horns were to enhance complicity in adhering to passively maintained territorial boundaries and/or resource allocation. While monogamous mating systems are often maintained to enhance the realized fecundity and fitness of both partners by decreasing partner search efforts and inter-batch timing [14,71], pairings may also be based on the benefits of co-operative behaviours [18,24,71]. For example, where paired individuals split foraging and vigilance roles to enhance protection against competitors and predators whilst maximizing food intake [13,14,21–24], males often devote proportionally more time to defending and maintaining territories so as females can forage more frequently to maximize reproductive output [14,24].

*H*. *varius* pairing in Kimbe Bay, Papua New Guinea was exclusively heterosexual and both size and, to a lesser degree, age assortative. This strongly suggests that pair formation has reproductive benefits and may also suggest some level of social monogamy. However, while long-term pair bonds are common among the Chaetodontidae family [70], this study represents only a single snapshot in time and direct evidence is required to verify that heterosexual pairs of *H*. *varius* do in fact represent socially and/or genetically monogamous couplings. Other studies suggest reproductive benefits may not necessarily be the primary driver of pair formation [13,14,18,24–27]. For example, pairing may potentially be driven firstly by other fitness benefits, such as joint territory defense, and heterosexual pairing and reproductive monogamy might be a secondary adaptation to this [24]. Selection for mates with larger horn structures would remain beneficial under this alternative scenario for pair formation, particularly if males do invest more energy in territorial protection [14,24].

In summary, this is the first record of sexual dimorphism in a pair forming reef fish. The results provide strong evidence for sexual dimorphism in horn and hump length, with emergence only after sexual maturation and increasing divergence as body size increased. We propose longer horns and humps in males of *H*. *varius* represent secondary sexual characteristics. The comparatively longer humps and horns among male members of heterosexual pairs provides further evidence to support the hypothesis that these head protrusions are utilized in sexual selection. Interestingly, while males consistently had larger horns than their female partners, there was no pattern in how much larger male horns should be. This represents a unique morphological characteristic among pair-forming reef fish and further investigation is warranted to establish the significance of larger head protrusions and their roles in partner choice and reproductive success.

## Supporting information

S1 Fig Map of Kimbe Bay on New Britain Island, Papua New Guinea. Circles show the location of the nine study sites.
(TIF)

S2 Fig Histological image of a female ovary (a) and male testes (b).
(TIF)

S3 Fig Sagittal otoliths of *Heniochus varius*. Used for determining annual age increments. Top: Male, Age = 4; Bottom: Female, Age = 7. Red dots indicate growth increments.
(TIF)

S1 Data LengthWeightAgeTL*Hvarius*. (CSV)

S2 Data HumpHornTL*Hvarius*. (CSV)

S3 Data HumpHornPairs*Hvarius*. (CSV)
