# Supplementary figures and images for: Sexual dimorphism in the horn size of a pair-forming coral reef butterflyfish
Source: PLoS One. 2020 Oct 8;15(10):e0240294. doi: 10.1371/journal.pone.0240294 (PMC7544049; doi:10.1371/journal.pone.0240294)

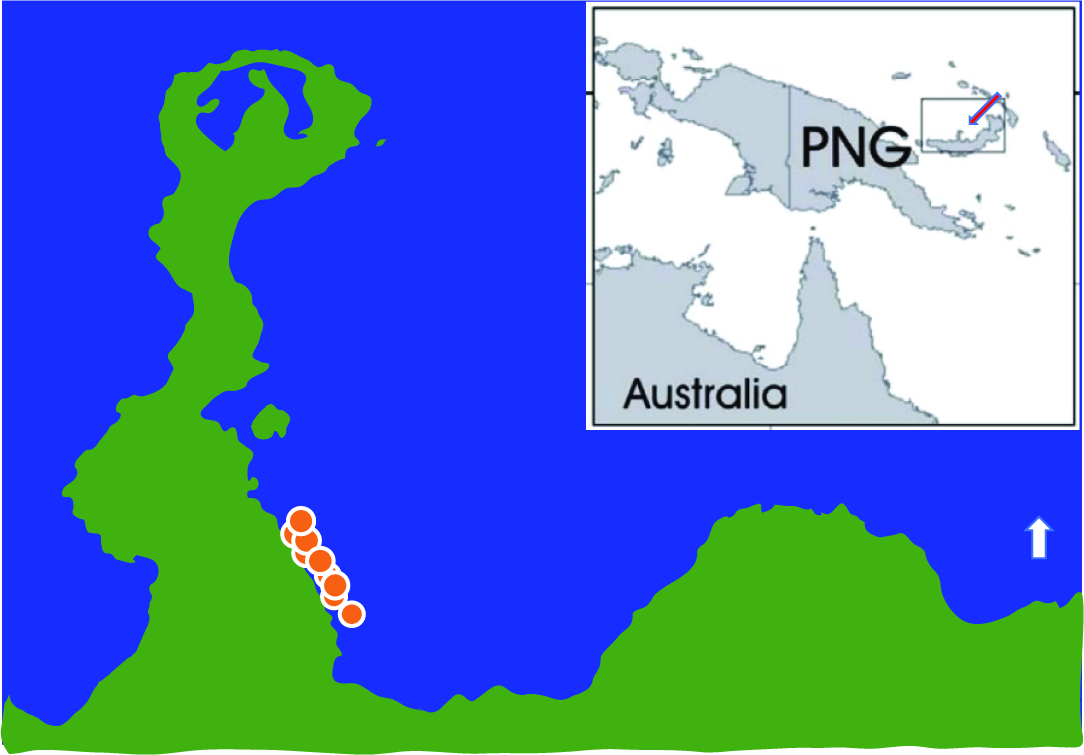

Supplement: S1 Fig — Circles show the location of the nine study sites. (TIF) [file pone.0240294.s001.tif]

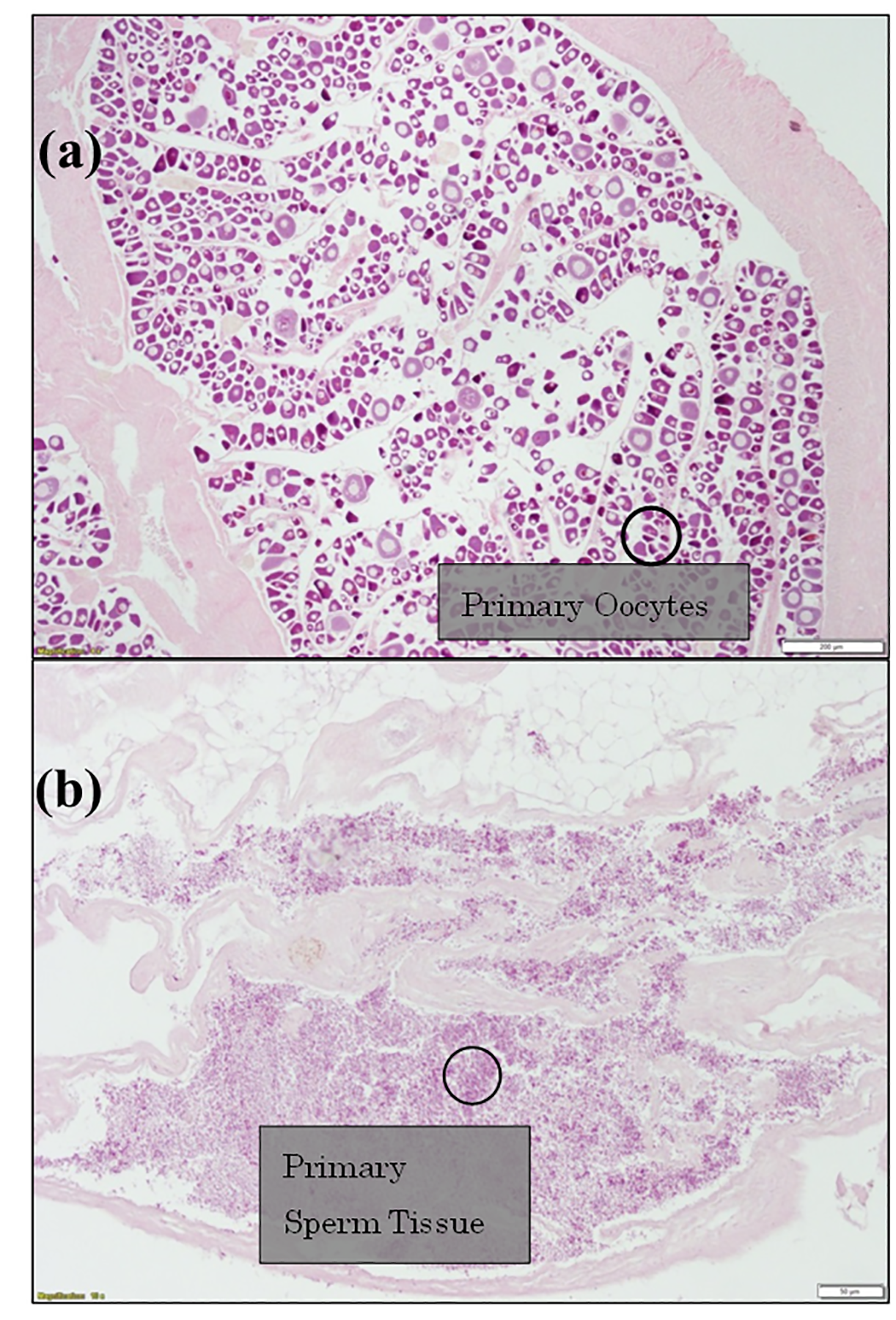

Supplement: S2 Fig — Histological image of a female ovary (a) and male testes (b). (TIF) [file pone.0240294.s002.tif]

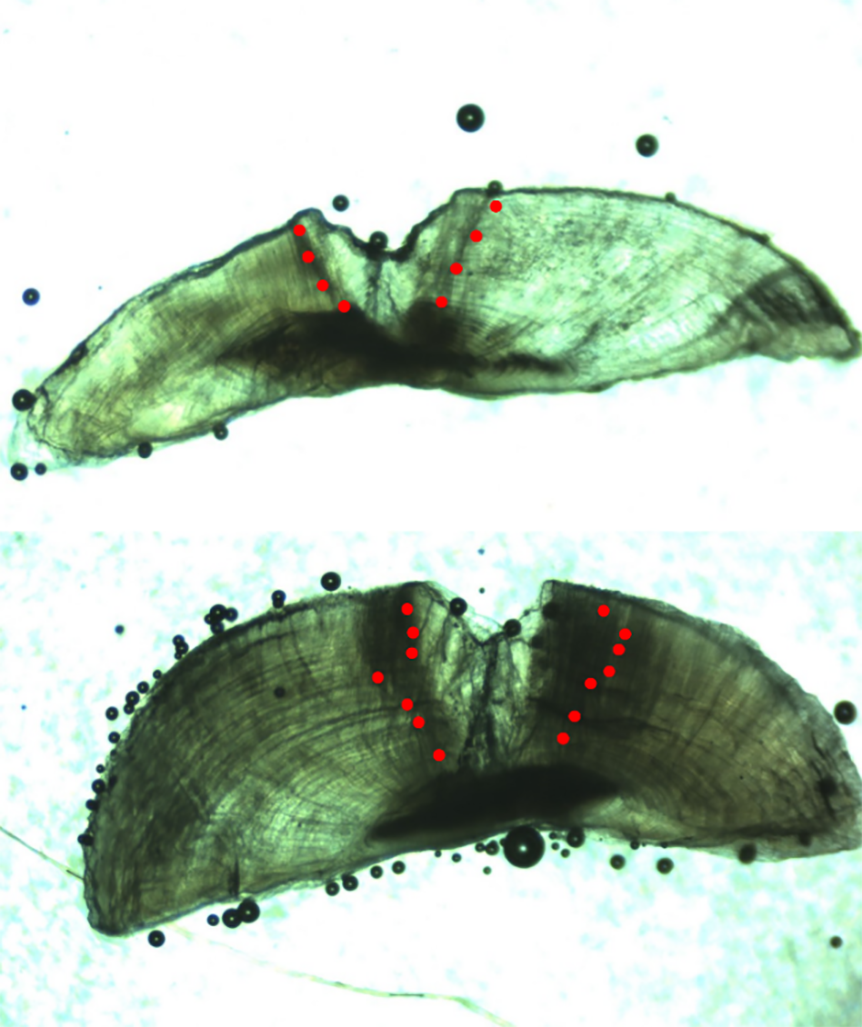

Supplement: S3 Fig — Used for determining annual age increments. Top: Male, Age = 4; Bottom: Female, Age = 7. Red dots indicate growth increments. (TIF) [file pone.0240294.s003.tif]
